# Fragility Induced by Interdependency of Complex Networks and Their Higher-Order Networks

**DOI:** 10.3390/e25010022

**Published:** 2022-12-23

**Authors:** Chengjun Zhang, Yi Lei, Xinyu Shen, Qi Li, Hui Yao, Di Cheng, Yifan Xie, Wenbin Yu

**Affiliations:** 1 School of Computer and Software, Nanjing University of Information Science and Technology, Nanjing 210044, China; 2 Engineering Research Center of Digital Forensics, Ministry of Education, Nanjing University of Information Science and Technology, Nanjing 210044, China; 3 Jiangsu Engineering Center of Network Monitoring, Nanjing University of Information Science and Technology, Nanjing 210044, China; 4 Jiangsu Collaborative Innovation Center of Atmospheric Environment and Equipment Technology (CI-CAEET), Nanjing University of Information Science and Technology, Nanjing 210044, China

**Keywords:** interdependent networks, higher-order organization, cascading failure, first-order phase transition

## Abstract

The higher-order structure of networks is a hot research topic in complex networks. It has received much attention because it is closely related to the functionality of networks, such as network transportation and propagation. For instance, recent studies have revealed that studying higher-order networks can explore hub structures in transportation networks and information dissemination units in neuronal networks. Therefore, the destruction of the connectivity of higher-order networks will cause significant damage to network functionalities. Meanwhile, previous works pointed out that the function of a complex network depends on the giant component of the original(low-order) network. Therefore, the network functionality will be influenced by both the low-order and its corresponding higher-order network. To study this issue, we build a network model of the interdependence of low-order and higher-order networks (we call it ILH). When some low-order network nodes fail, the low-order network’s giant component shrinks, leading to changes in the structure of the higher-order network, which further affects the low-order network. This process occurs iteratively; the propagation of the failure can lead to an eventual network crash. We conducted experiments on different networks based on the percolation theory, and our network percolation results demonstrated a first-order phase transition feature. In particular, we found that an ILH is more fragile than the low-order network alone, and an ILH is more likely to be corrupted in the event of a random node failure.

## 1. Introduction

**Figure 1:**
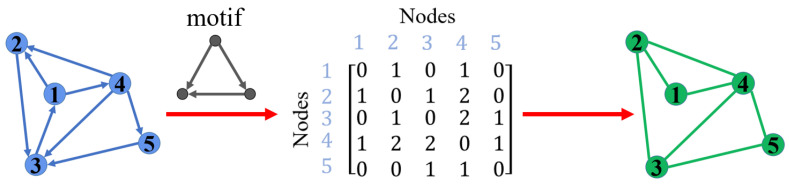
In a directed network with 5 nodes, an adjacency matrix can be generated by calculating the co-occurrence times of two nodes in the motif, and then the corresponding higher-order network can be obtained by using the adjacency matrix.

The study of complex networks involves many fields, such as the Internet, social networks, power networks, and transportation networks. In the past two decades, research on complex networks has mainly focused on real-world networks [1,2,3,4,5,6,7,8], these original networks can also be viewed as low-order networks. In recent years, higher-order networks have begun to attract more and more researchers’ attention [9]. The generation of higher-order networks is based on network motifs. A network motif is a network subgraph composed of three or more nodes, it is the basic unit for building complex networks, and it is also a valuable structure for implementing network functions [10,11,12]. One can generate a corresponding higher-order network based on the original network through a specific network motif. A specific demonstration is shown in Figure 1. Given a network and a motif, the framework generates an adjacency matrix by calculating the number of times two nodes co-occur in the motif. Then, an undirected higher-order network can be generated based on this adjacency matrix. Changes in the low-order structure will affect the higher-order structure, because changes in the low-order structure will affect the way the triangle is connected [9]. However, in the international trade network, the higher-order structure corresponds to an alliance. Suppose a node in an alliance withdraws from the alliance, the alliance may also collapse, and each node in the alliance cannot conduct trade through the alliance. Therefore, in the corresponding higher-order network, if a triangle in the high-order network is destroyed, the triangle will disappear in the higher-order network, and the corresponding edge in the low-order network will also be destroyed. Studying the characteristics of higher-order networks can help researchers identify important nodes in the network and then protect these important nodes through special means [13]. For instance, researchers used higher-order networks to study the spread of pollutants in the air and then provided valuable recommendations for environmental governance [14]. Therefore, it is essential to study the properties of higher-order networks, which can help us deeply explore the properties and the dynamic behavior of networks [15,16].

In complex networks, the robustness of the network is an essential issue. A significant number of traditional studies focused on the robustness of original (low-order) networks [17,18,19,20]. The robustness of a network is usually determined by the giant component of the network, which is the one with the largest size among all connected components in the network. If the giant component of the network is compromised, the functionality of the network can be significantly affected [21]. In reality, many real-world network systems exhibit the “scale-free” property; these networks typically feature a solid tolerance to random attacks, but they are vulnerable to deliberate attacks [22,23]. In the economic field, studying the robustness of the network can help us discover the risks existing in the economic system [24]. In the infrastructure network, the stability of the infrastructure can be evaluated by studying the network robustness, and then a more robust infrastructure network can be designed [25]. On the Internet, studying the robustness of the network can improve network safety [26]. On the other hand, studies have found that networks exhibit rich higher-order structures, and the connectivity patterns of these higher-order structures play a crucial role in understanding and controlling many complex systems [9]. For example, higher-order organizations composed of open bidirectional wedges are significant for brain neural networks [27]; higher-order structures composed of triangular motifs also play a crucial role in social networks [28]. Therefore, it is fair to say that higher-order networks reveal structural patterns in complex systems, and if higher-order networks are compromised, the functionality of the entire network will be inevitably damaged.

To address this issue, we propose a network model in which both higher-order and low-order networks are interdependent. We conduct experiments on real-world networks and apply percolation theory to study the robustness of these networks based on this model. Theoretically, we find that first-order phase transitions characterize the network percolation results for this network model. In application, our model proves that when the low-order network and its higher-order network are coupled, the network will be more fragile.

## 2. Related Works

Traditional research on the robustness of complex networks generally focuses on the low-order (original) networks, and the robustness of a network typically refers to the network’s ability to maintain its connectivity when some nodes or edges are damaged. The robustness of the network can be expressed by the size of its largest connected component after being attacked [29,30]. In 2000, Albert et al. found that many networks with scale-free properties had high robustness [22]. In another study in 2007, Karrer et al. assessed the importance of community structure by measuring the robustness of the network. They proposed a suitable method of perturbing the network, studied the changes in network structure after perturbation, and used them to determine the importance of community structure [31]. Furthermore, Peng et al. in 2016 showed a conflicting relationship between the small-world effect and network robustness when the degree distribution of the network was the same. Increasing the robustness of the network could reduce the small-world effect. They proposed an optimization model to obtain an optimal trade-off between small-world effect and network robustness [32]. In addition, in 2020, Smolyak et al. proposed a method for protecting critical nodes to mitigate cascading failures and validated it on a financial network [33]. In summary, the study of network robustness remains a crucial research direction in complex networks.

To reveal the structural design principles of complex networks, Milo et al. defined “network motifs” in 2002 based on collecting a large amount of data and conducting many analyses. Some interconnection patterns frequently appear in complex networks, and the number of such patterns is higher than in random networks [34]. Network motifs affect network functioning. For example, in the central neural network of the brain, open bidirectional wedges play a crucial role, and triangular motifs frequently appear in social networks. The most subsequent studies are based on the statistics of network motifs.

Over the years, researchers have explored the influence of motif structure on complex networks. In 2016, Benson stated that complex networks could represent rich higher-order organizations through different network motifs, and they could be displayed based on the clustering of higher-order organizations. The authors proposed the theoretical knowledge of higher-order networks [9]. Additionally, real-world problems can be analyzed and solved using the knowledge of the higher-order organization of complex networks. In 2017, Wang et al. applied higher-order network methods to study the generation and propagation paths of PM2.5 in urban networks [14]. In 2021, Xia et al. used different attack strategies on the original network and then observed the robustness of the low- and higher-order networks. They found that higher-order networks were more fragile than original networks [35]. Hypergraphs can be used to explore higher-order structures and capture higher-order interactions in complex systems. In 2022, Peng Hao et al. studied the robustness of the hypergraph network and found that the network will be more fragile when the probability of large-cardinality hyperedges and high-degree nodes, being deleted, increases [36,37]. Exploring the properties of higher-order networks is becoming increasingly important for the study of complex networks.

Many studies in the field of complex networks have analyzed individual networks, meaning that the interaction between different networks has been ignored. However, many networks in the real world are interdependent and interact with each other [38,39,40,41]. In 2010, Buldyrev et al. established a model of interdependent networks, and their research showed that in interdependent networks, networks with a wider degree distribution are more vulnerable to general attacks. In addition, the authors gave an exact analytical solution to the first-order phase transitions [42] in interdependent networks [43]. Furthermore, in 2013, Huang et al. devised an analytical approach to study the impact of clustering within an interdependent network on network robustness. Their results suggested that clustering increased the network’s vulnerability [44]. In 2016, Sun et al. found that degree heterogeneity had a greater impact on the vulnerability of interdependent networks; their research showed that a stronger coupling between networks led to a more fragile network. Last but not least, in 2021, Turalska et al. proposed two control strategies to control cascading failures in interdependent networks [45].

Higher-order networks can realize the functions of networks. Therefore, when we study complex networks, we should not focus only on low-order networks but rather organically combine high- and low-order networks for analysis. As the function of a network not only depends on the low-order network but also the higher-order network, we build a network model, in which the low- and higher-order networks are interdependent. We study in this paper the properties of this interdependent network, such as the phase transition type of network percolation and the robustness of the network under attack.

## 3. Methods and Data

### 3.1. Methods

**Figure 2:**
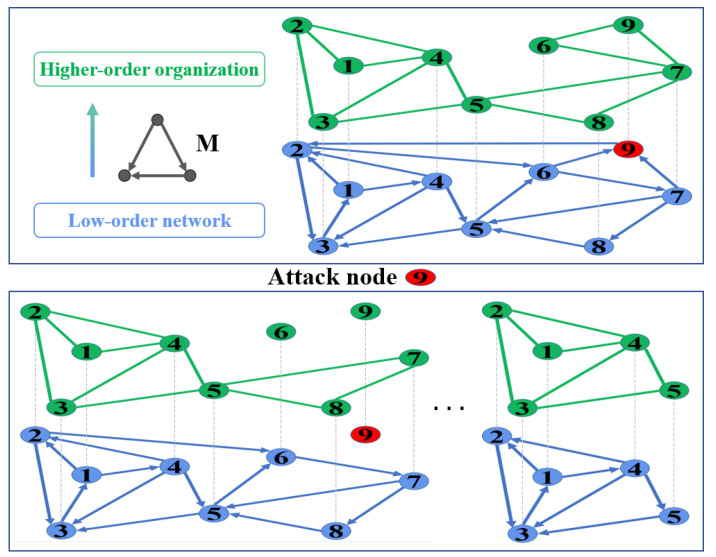
The ILH model. The original low-order network can generate a corresponding higher-order network through the three-node motif M (upper panel), and the cascading failure occurs in ILH (bottom panel).

If a directed network is strongly connected, with the help of the theory of higher-order networks, we can generate a higher-order network corresponding to the low-order network through a certain three-node network motif M. We propose a model of the interdependence of complex networks and their higher-order networks, as shown in Figure 2. Some nodes in the low-order network are attacked or fail, and we simulate these nodes failure by removing them. For example, if node 9 is removed from the low-order network, then nodes 9 and 6 in the higher-order network generated by the low-order network will fail, causing node 6 in the low-order network to fail. At this time, nodes 7 and 8 do not belong to the giant strongly connected component of the low-order network, so they will be removed from the low-order network, causing the failure of nodes 7 and 8 in the higher-order network. In this way, the whole interdependent network is stable, and only five nodes remain in both the low- and higher-order networks. The entire simulation process is the case of ILH cascading failures.

Our research focuses on directed networks. Based on the percolation theory, when a network fails, it will split into multiple connected components with different scales, among which the giant strongly connected component has the largest scale, able to retain network functions. We randomly remove 1−p nodes from the network to simulate cascading failures in the network. When the remaining *p* nodes in the network reach the critical value of $p_{c}$, the network gains giant strongly connected components. The term P∞ represents the ratio of the number of nodes $N^{\prime }$ in the giant strongly connected component to the total number of nodes *N*:

(1) $$P\infty =\frac{N^{\prime }}{N}$$

### 3.2. Data Description

In this paper, we first use three undirected random networks, Erdős–Rényi networks(ER) [46,47], Scale-Free networks(BA) [48,49], where λ = 2.6, Small-World networks (SW) [50], which has a rewiring probability of 0.05. Then, we randomly specify a certain direction for each undirected edge with a probability 0.5. Therefore, these three types of undirected networks will become directed networks; the ER, SW, and BA mentioned below all refer to the corresponding directed networks. Here, we do not take bidirectional edges into consideration because the number of bidirectional edges in real-world networks is small; for example, the proportion of bidirectional edges in CELEGANS is only 8.4%, and only 6.9% in CHESS. If no description is given, the number of random network nodes generated is 1000 by default. To study the evolution of real-world networks, we then analyzed 14 directed networks, which are CELEGANS [50], EMAIL, GD06, TRUST, SPAM, PAIRS, PAGES, CHESS, CORA [51], POLBLOGS [52], UTM1700, MARAGAL, UTM3060 [53], ODLIS [54], respectively. PAGES, EMAIL, TRUST, POLBLOGS are social networks. CORA is the scientific paper citation network. Nodes in GD06 represent classes in Java, and edges represent dependencies between classes. SPAM is a network of hyperlinks to pages. CELEGANS is a neural network. In the CHESS network, nodes represent players, and edges represent two players in a chess match. ODLIS is an online dictionary network. In the PAIRS network, nodes represent words, and edges represent associated words associated with words. UTM1700, UTM3060, MARAGAL are miscellaneous networks of downloads in the internet. Table 1 gives some properties of these networks. Since some networks are not strongly connected, we use the giant strongly connected component of the network for experiments.

## 4. Experiments and Results

**Figure 3:**
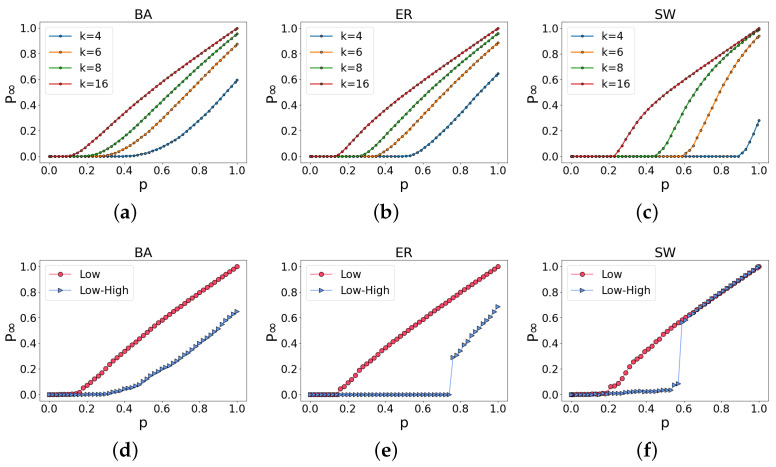
Robustness of low-order networks and the robustness of ILH: (**a**–**c**) are the robustness of random networks generated according to different average degrees <k>. Obviously, the greater the average degree of the network is, the higher the robustness of the network is. In (**d**–**f**), the average degree <k> of the low-order network is 16 and the robustness of ILH is lower than that of low-order networks. In addition, in the ER and SW networks, the percolation of ILH exhibits a first-order phase transition.

In this paper, we study the robustness of low-order networks for comparison and then the robustness of an ILH. As clearly shown in Figure 3a–c, under the same network size, networks with different average degrees <k> have different robustness. Regardless of the network type, a larger average degree implies more redundant wiring of the network. In other words, additional paths between two nodes exist in the network, enhancing the network’s connectivity. Even when some nodes are removed, the network still has high robustness. Next, we simulate a random attack on the ILH, and the average degree <k> of the low-order network is 16, as shown in Figure 3d–f. In the ER and SW networks, unlike the percolation of the low-order network (denoted by “Low” in these images), the percolation of ILH (“Low–High”) is characteristic of a first-order phase transition. Still, in the SW network, this phase transition is intermittent. However, in the BA network, both the percolation of the low-order network and that of the ILH exhibit second-order phase transitions. From the results, the robustness of ILH is clearly lower than that of low-order networks regardless of the random network; i.e., ILH is relatively fragile.

**Figure 4:**
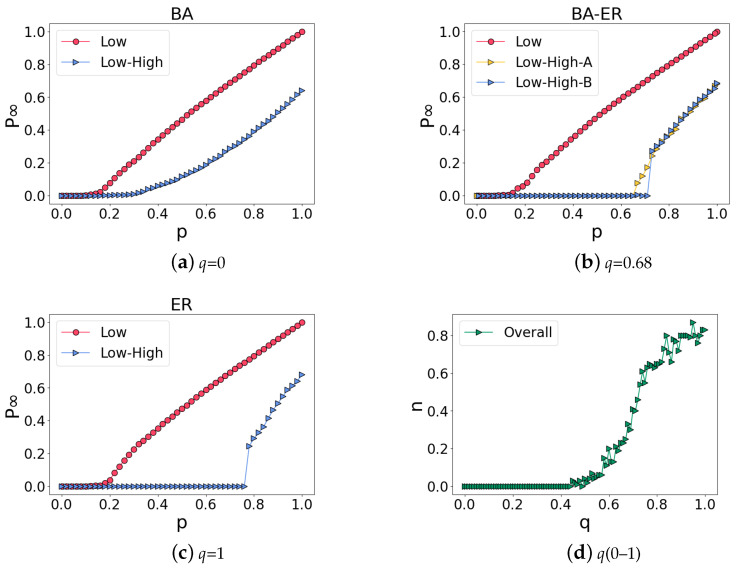
Percolation of ILH in which the low-order network changes gradually from a BA network to an ER network by adjusting the link rewiring probability *q*. For comparison, the percolation on the low-order network alone is shown. (**a**–**c**) show the phase transition of the percolation in networks with *q* = 0, *q* = 0.68 and *q* = 1, respectively. In (**b**), we can observe two kinds of phase transitions, second-order phase transition (Low-High-A) and first-order phase transition (Low-High-B) in the percolation of ILH; these two kinds of phase transitions occur with different probabilities and the probability is demonstrated in (**d**). While the percolation always exhibits the second-order phase transition in the low-order network alone, the percolation phase transition of ILH becomes the first-order when *q* is large. (**d**) shows the probability *n* of observing the first-order phase transition under different link rewiring probability *q* in ILH.

We observe that the percolation of the BA network shows all second-order phase transitions, so we rewire the BA network and disconnect the edges of the network with a ratio of *q*. Then, we randomly select two nodes without edges from the network to add a directed edge to them until the number of network edges returns to the initial state. Finally, we simulate a random attack on ILH, and the results are shown in Figure 4a–c. When q=0, the network is the BA network; when q=1, the network becomes an ER network. In the process of increasing *q* from 0 to 1, the percolation of ILH gradually changes from a second-order to a first-order phase transition. For example, when q=0.68, the percolation of ILH changes from the second-order to the first-order phase transition. However, this does not mean that the rewired network will show a first-order phase transition at every test at q=0.68. After 100 tests, the number of first-order phase transitions in the percolation of ILH at various *q* values are shown in Figure 4d. When *q* increases from 0 to 1, the network gradually changes from a BA network to ER network, and the probability *n* of first-order phase transition in the percolation of ILH continues to increase.

**Figure 5:**
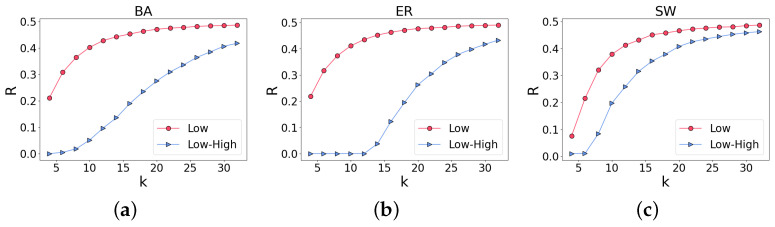
Robustness of low-order networks and ILH under different average degrees, (**a**–**c**) are the cases of BA, ER, and SW respectively, where *R* is the area under the curve when P∞ and *p* change. As the average degree <k> increases, *R* increases, which means that the robustness of the network is enhanced. As these results show, the robustness of ILH is lower than that of low-order networks.

The above results preliminarily demonstrate that in some networks, such as ER, the percolation of the ILH may indicate a first-order phase transition. Next, we study the robustness of the ILH. Using the change of $P_{\infty }$ with the parameter *p* mentioned above, we can calculate the area *R* under the curve and determine the network robustness by comparing the areas. A larger *R* indicates a more robust network. Likewise, a smaller *R* means a less robust network. As shown in Figure 5, different random networks are studied, observing the robustness of low-order networks and that of ILH by adjusting the average degree of the networks. The figure shows that ILH are significantly more vulnerable than low-order networks. However, this method compares the overall network performance, i.e., macro performance. A significant feature of interdependent networks is that when a node in the network fails, recursive failures of interconnected nodes result in other networks, leading to large-scale failures. Our results are in good agreement with this conclusion. An ILH is too fragile compared to low-order networks, although this difference in vulnerability can be compensated as the average degree of the network increases. However, an increase in the average degree yields additional edges of the network, which largely increases the cost of the network.

**Figure 6:**
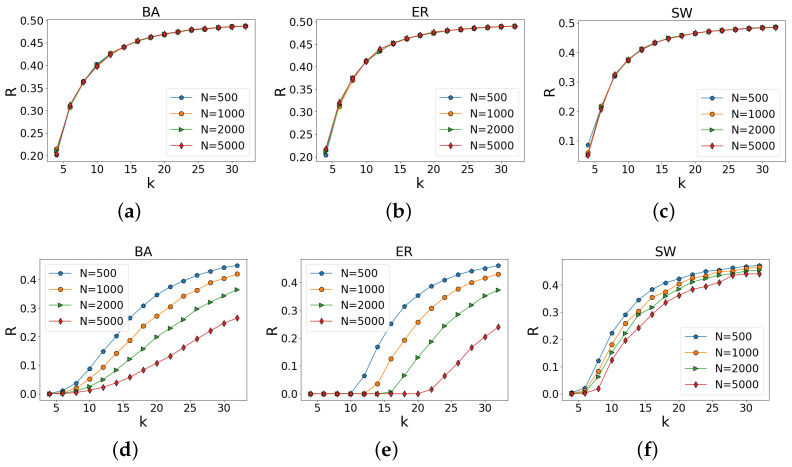
Effect of network size on network robustness, (**a**–**c**) are low-order networks; obviously, when the average degree <k> of the network is the same, the network size *N* has little effect on the robustness of the network. (**d**–**f**) show the effect of network size on the robustness of ILH; results show that when the average degree <k> is the same, the larger the size *N* of the network is, and the lower the robustness of ILH is.

**Figure 7:**
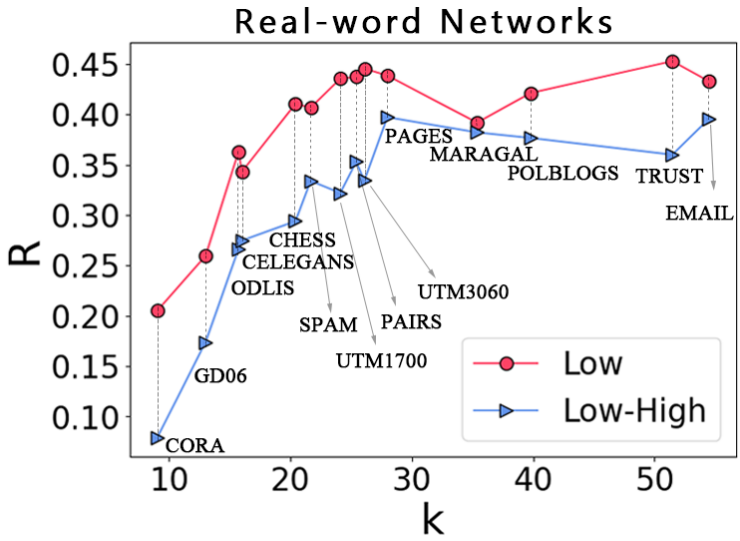
Robustness of low-order networks and ILH on empirical networks. It can be observed from the results that the robustness of ILH is lower than that of low-order networks; this means that ILH are more vulnerable than low-order networks.

Our analysis shows that the percolation of an ILH on some random networks will show a first-order phase transition and that an ILH is more fragile than low-order networks. Thus, we further ask, what effect do different network sizes have on the robustness of complex networks? As shown in Figure 6a–c, when the average degree of the network is the same, the scale of the network has little effect on the robustness of the low-order network. However, in Figure 6d–f, when the average degree <k> is the same, the larger the scale of the network, the more vulnerable the ILH. This means that ILH is also affected by the size of the network; such a conclusion strongly indicates that ILH is more vulnerable, given that the real-world networks are usually of large size. Finally, we use the results obtained on 14 empirical networks as a concluding work. We calculate the area under the curves obtained when percolation occurred for each network separately and aggregated the results. As shown in Figure 7, among these different types of networks, no matter if it is the social network POLBLOGS or the neural network CELEGANS, the low-order network is more robust than the ILH, which also means that ILH is more fragile.

## 5. Conclusions and Discussion

The network motif is the basic building unit of the complex network, and the higher-order network generated by the motif can describe the internal structure of the complex network well. Motifs and higher-order networks play crucial roles in understanding many complex systems [9,10,34]. The low- and higher-order networks should be organically combined to form an interdependent network; therefore, the study of the ILH is of great significance. In this paper, we propose a model of the interdependence of complex networks and their higher-order networks. When some nodes in the low-order network fail, a failure of the dependent nodes may result in the failure of nodes in the corresponding higher-order network. Such failures can occur recursively, owing to the interaction of nodes between the networks, and leading to a chain of failures. Our results also demonstrate that the percolation of an ILH undergoes a first-order phase transition (i.e., discontinuous transition), which is different from the second-order phase transition that occurs with the percolation of a single network. In addition, the ILH will be more vulnerable and more likely to be paralyzed when it fails or is attacked.

In the future, one may consider building multi-layered interdependent networks of multiple motifs. At present, only the two-layer interdependent network is studied. One can try two kinds of motifs, generate two corresponding higher-order networks, and then couple them with the original low-order network to form a three-layer network. One can also aim to determine which nodes should be protected in this multi-layer network to avoid network damage. In addition, only directed networks are discussed in this work; undirected networks also need to be investigated. We hope that our research can provide inspiration for a wide range of scholars to contribute to the development of low-high order interdependent networks.

## Figures and Tables

**Table 1:** Statistical properties of the empirical networks, where *N* is the number of nodes, *M* is the number of edges, <k> is the average degree of the network, <d> is the average shortest path of the network, *C* is the network clustering coefficient, and *r* is the degree assortativity.

Networks	*N*	*M*	<k>	<d>	*C*	*r*
CELEGANS	297	2345	15.79	4.00	0.17	−0.26
EMAIL	906	12,085	26.68	2.68	0.34	0.08
POLBLOGS	1224	19,022	31.08	3.19	0.22	−0.19
GD06	1538	8032	10.44	5.21	0.22	−0.12
UTM1700	1700	19,809	23.30	11.25	0.41	0.33
MARAGAL	1964	26,692	27.18	3.23	0.10	−0.14
ODLIS	2900	18,241	12.58	4.59	0.18	0.01
UTM3060	3060	39,151	25.59	14.43	0.39	0.34
TRUST	4658	40,133	17.23	2.90	0.09	0.11
SPAM	4767	37,375	15.68	3.81	0.14	0.04
PAIRS	5018	63,608	25.35	4.26	0.13	−0.02
PAGES	7057	89,429	25.34	4.25	0.21	0.07
CHESS	7301	60,046	16.45	4.29	0.10	0.39
CORA	23,166	91,500	7.90	13.33	0.15	0.02

## Data Availability

All data are presented in main text.
